# 4-Chloro-2′,4′,6′-triethyl­benzophenone: a redetermination

**DOI:** 10.1107/S1600536811009639

**Published:** 2011-03-19

**Authors:** Hiroki Takahashi

**Affiliations:** a Graduate School of Human and Environmental Studies, Kyoto University, Kyoto 606-8501, Japan

## Abstract

The structure of the title compound [systematic name: (4-chloro­phen­yl)(2,4,6-trimethyl­phen­yl)methanone], C_19_H_21_ClO, has been redetermined at 100 K. The redetermination is of significantly higher precision than the previous structure determination at 133 K and reveals disorder of the one of the *o*-ethyl groups [occupancy factors = 0.77 (1) and 0.23 (1)] that was not identified in the previous report [Takahashi & Ito (2010 ▶). *CrystEngComm*, **12**, 1628–1634]. The C—C—C—C torsion angles of the major and minor disorder components of the ethyl group with respect to the attached benzene ring are −103.7 (2) and −172.0 (6)°, respectively. It is of inter­est that the title compound does not display a single-crystal-to-single-crystal polymorphic phase transition on cooling, as was observed for a closely related compound, a fact that can be attributed to the disorder in the ethyl group.

## Related literature

For the structure of the title compound at 133 K and the phase transition observed in a related compound, see: Takahashi & Ito (2010 ▶). For its solid-state photochemical properties, see: Ito *et al.* (2009 ▶). For the synthesis, see: Ito *et al.* (1985 ▶).
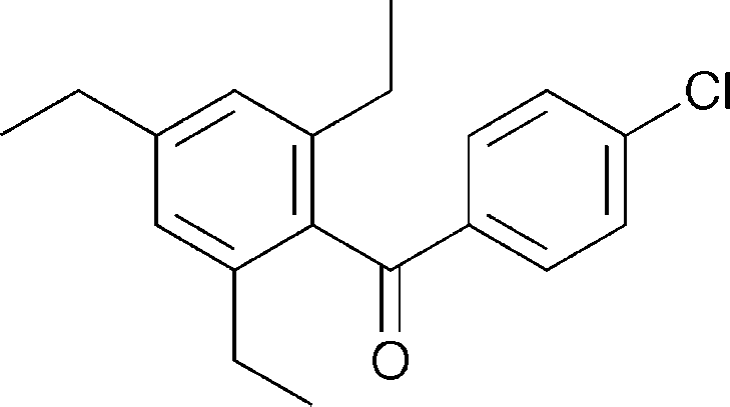


## Experimental

### 

#### Crystal data

C_19_H_21_ClO
*M* *_r_* = 300.81
Monoclinic, 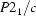

*a* = 10.3329 (6) Å
*b* = 15.8383 (8) Å
*c* = 10.6876 (6) Å
β = 111.0116 (16)°
*V* = 1632.78 (16) Å^3^
*Z* = 4
Mo *K*α radiation
μ = 0.23 mm^−1^
*T* = 100 K
0.35 × 0.27 × 0.20 mm

#### Data collection

Rigaku R-AXIS RAPID diffractometer
Absorption correction: multi-scan (*ABSCOR*; Higashi, 1995 ▶) *T*_min_ = 0.745, *T*_max_ = 1.000
15675 measured reflections
3738 independent reflections
3287 reflections with *I* > 2σ(*I*)
*R*_int_ = 0.032

#### Refinement

*R*[*F* ^2^ > 2σ(*F* ^2^)] = 0.040
*wR*(*F* ^2^) = 0.104
*S* = 1.08
3738 reflections
264 parameters
H atoms treated by a mixture of independent and constrained refinement
Δρ_max_ = 0.43 e Å^−3^
Δρ_min_ = −0.19 e Å^−3^

### 

Data collection: *RAPID-AUTO* (Rigaku, 1998 ▶); cell refinement: *RAPID-AUTO*; data reduction: *Yadokari-XG 2009* (Kabuto *et al.*, 2009 ▶); program(s) used to solve structure: *SIR97* (Altomare *et al.* (1999 ▶); program(s) used to refine structure: *SHELXL97* (Sheldrick, 2008 ▶); molecular graphics: *Yadokari-XG 2009* and *ORTEP-3* (Farrugia, 1997 ▶); software used to prepare material for publication: *Yadokari-XG 2009* and *publCIF* (Westrip, 2010 ▶).

## Supplementary Material

Crystal structure: contains datablocks I, global. DOI: 10.1107/S1600536811009639/sj5114sup1.cif

Structure factors: contains datablocks I. DOI: 10.1107/S1600536811009639/sj5114Isup2.hkl

Additional supplementary materials:  crystallographic information; 3D view; checkCIF report

## Figures and Tables

**Fig. 1.**
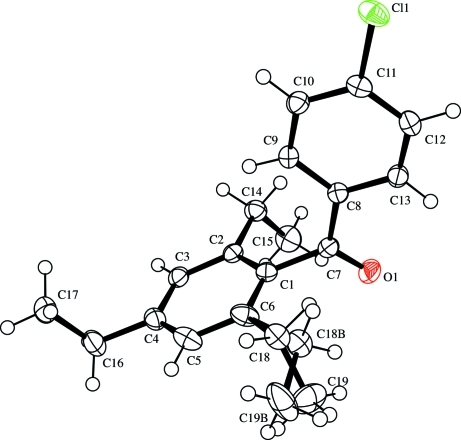
The structure of the title compound with ellipsoids drawn at the 50% probability level and the atom numbering scheme.

**Fig. 2.**
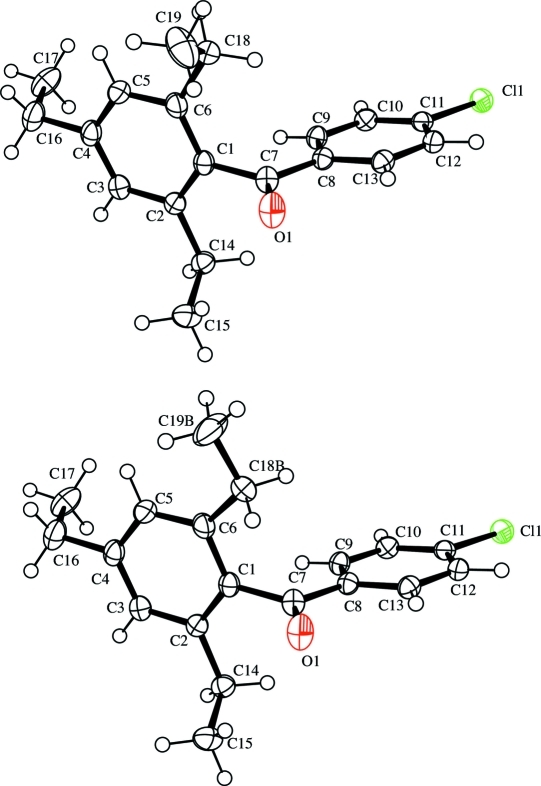
The structure of the title compound with ellipsoids at the 50% probability level showing the major occupancy molecule (top) and the minor one (bottom).

## supplementary crystallographic information

### Comment

The title compound, 4-chloro-2',4',6'-triethylbenzophenone, is  analogous to 3,4-dichloro-2',4',6'-triethylbenzophenone that undergoes a single-crystal-to-single-crystal polymorphic phase transition on cooling the crystal to 166 K (Takahashi & Ito 2010). In this phase transition one of the *o*-ethyl groups rotates by 180 °.

Crystal structures of the title compound at 133 K and 173 K were already reported (Takahashi & Ito, 2010: Ito *et al.* 2009). The crystal structure of the title compound has been redetermined at 100 K. This crystal does not show the same phase transition in this temperature range. However in this structure, one of *o*-ethyl groups was disordered over two positions with a site-occupancy ratio of 0.77 (1) and 0.23 (1). The molecular structure of the title compound is shown in Fig. 1. The dihedral angles of the C1—C6—C18—C19 (major disorder component) and C1—C6—C18B—C19B (minor component) are -103.7 (2) and -172.0 (6) °, respectively. This disordered ethyl group operates as a buffer in the crystal on shrinking the crystal lattice, hence this compound does not show the phase transition at low temperature.

### Experimental

The title compound was prepared from 1,3,5-triethylbenzene and 4-chlorobenzoyl chloride by a Friedel-Crafts reaction as described in the literature (Ito *et al.* 1985). Colourless prism-like crystals were obtained by slow evaporation of an MeOH solution of the title compound.

### Refinement

The H atoms of the disordered ethyl groups and the methyl group in the *p*-ethyl substituent in the molecule were positioned with idealized geometry using a riding model with C—H = 0.98 Å. All other H atoms were refined with isotropic displacement parameters (set to 1.2 or 1.5 times the *U*_eq_ of the parent atom).

### Crystal data

**Table:**

C_19_H_21_ClO	*F*(000) = 640
*M**_r_* = 300.81	*D*_x_ = 1.224 Mg m^−^^3^
Monoclinic, *P*2_1_/*c*	Mo *K*α radiation, λ = 0.71075 Å
Hall symbol: -P 2ybc	Cell parameters from 12293 reflections
*a* = 10.3329 (6) Å	θ = 3.3–27.5°
*b* = 15.8383 (8) Å	µ = 0.23 mm^−^^1^
*c* = 10.6876 (6) Å	*T* = 100 K
β = 111.0116 (16)°	Prism, colourless
*V* = 1632.78 (16) Å^3^	0.35 × 0.27 × 0.20 mm
*Z* = 4	

### Data collection

**Table:**

Rigaku R-AXIS RAPID diffractometer	3738 independent reflections
Radiation source: sealed X-ray tube	3287 reflections with *I* > 2σ(*I*)
Detector resolution: 10.00 pixels mm^-1^	*R*_int_ = 0.032
ω scans	θ_max_ = 27.5°, θ_min_ = 3.3°
Absorption correction: multi-scan (*ABSCOR*; Higashi, 1995)	*h* = −13→13
*T*_min_ = 0.745, *T*_max_ = 1.000	*k* = −20→20
15675 measured reflections	*l* = −12→13

### Refinement

**Table:**

Refinement on *F*^2^	Primary atom site location: structure-invariant direct methods
Least-squares matrix: full	Secondary atom site location: difference Fourier map
*R*[*F*^2^ > 2σ(*F*^2^)] = 0.040	Hydrogen site location: inferred from neighbouring sites
*wR*(*F*^2^) = 0.104	H atoms treated by a mixture of independent and constrained refinement
*S* = 1.08	*w* = 1/[σ^2^(*F*_o_^2^) + (0.0576*P*)^2^ + 0.3777*P*] where *P* = (*F*_o_^2^ + 2*F*_c_^2^)/3
3738 reflections	(Δ/σ)_max_ = 0.001
264 parameters	Δρ_max_ = 0.43 e Å^−^^3^
0 restraints	Δρ_min_ = −0.19 e Å^−^^3^

### Special details

**Table:**

Geometry. All e.s.d.'s (except the e.s.d. in the dihedral angle between two l.s. planes) are estimated using the full covariance matrix. The cell e.s.d.'s are taken into account individually in the estimation of e.s.d.'s in distances, angles and torsion angles; correlations between e.s.d.'s in cell parameters are only used when they are defined by crystal symmetry. An approximate (isotropic) treatment of cell e.s.d.'s is used for estimating e.s.d.'s involving l.s. planes.
Refinement. Refinement of *F*^2^ against ALL reflections. The weighted *R*-factor *wR* and goodness of fit *S* are based on *F*^2^, conventional *R*-factors *R* are based on *F*, with *F* set to zero for negative *F*^2^. The threshold expression of *F*^2^ > σ(*F*^2^) is used only for calculating *R*-factors(gt) *etc*. and is not relevant to the choice of reflections for refinement. *R*-factors based on *F*^2^ are statistically about twice as large as those based on *F*, and *R*- factors based on ALL data will be even larger.

### Fractional atomic coordinates and isotropic or equivalent isotropic displacement parameters (Å^2^)

**Table:**

	*x*	*y*	*z*	*U*_iso_*/*U*_eq_	Occ. (<1)
C1	0.76918 (13)	0.22501 (7)	0.03078 (12)	0.0238 (2)	
C2	0.85966 (12)	0.16627 (7)	0.00844 (11)	0.0225 (2)	
C3	0.80584 (13)	0.08879 (8)	−0.04900 (12)	0.0252 (3)	
H1	0.8691 (16)	0.0474 (9)	−0.0616 (15)	0.029 (4)*	
C4	0.66661 (14)	0.06943 (8)	−0.08394 (12)	0.0284 (3)	
C5	0.57933 (14)	0.12902 (8)	−0.06129 (15)	0.0327 (3)	
H2	0.483 (2)	0.1172 (11)	−0.0864 (18)	0.044 (5)*	
C6	0.62763 (14)	0.20723 (8)	−0.00505 (15)	0.0313 (3)	
C7	0.82387 (13)	0.30863 (8)	0.09536 (12)	0.0254 (3)	
C8	0.82047 (12)	0.38213 (7)	0.00704 (12)	0.0223 (2)	
C9	0.79614 (13)	0.37152 (8)	−0.12921 (12)	0.0242 (2)	
H3	0.7853 (15)	0.3187 (10)	−0.1672 (15)	0.028 (4)*	
C10	0.79308 (13)	0.44082 (8)	−0.20960 (12)	0.0255 (3)	
H4	0.7778 (15)	0.4335 (10)	−0.3016 (16)	0.028 (4)*	
C11	0.81309 (12)	0.52059 (7)	−0.15245 (12)	0.0246 (2)	
C12	0.83723 (13)	0.53293 (7)	−0.01726 (13)	0.0254 (2)	
H5	0.8489 (16)	0.5894 (10)	0.0190 (16)	0.032 (4)*	
C13	0.84213 (12)	0.46330 (7)	0.06203 (12)	0.0243 (2)	
H6	0.8607 (15)	0.4687 (9)	0.1571 (15)	0.025 (3)*	
C14	1.01239 (13)	0.18466 (8)	0.04787 (13)	0.0277 (3)	
H7	1.0262 (17)	0.2453 (11)	0.0360 (16)	0.035 (4)*	
H8	1.0473 (16)	0.1530 (10)	−0.0154 (16)	0.035 (4)*	
C15	1.09458 (16)	0.16062 (10)	0.19341 (15)	0.0377 (3)	
H9	1.0600 (19)	0.1925 (12)	0.2570 (19)	0.050 (5)*	
H10	1.0850 (18)	0.1004 (11)	0.2077 (18)	0.042 (5)*	
H11	1.193 (2)	0.1717 (12)	0.2182 (19)	0.049 (5)*	
C16	0.60860 (17)	−0.01351 (9)	−0.15049 (15)	0.0385 (3)	
H12	0.5354 (19)	−0.0318 (11)	−0.1218 (17)	0.043 (5)*	
H13	0.681 (2)	−0.0566 (12)	−0.1199 (19)	0.049 (5)*	
C17	0.55351 (17)	−0.00718 (11)	−0.30280 (15)	0.0431 (4)	
H14	0.4820	0.0367	−0.3316	0.065*	
H15	0.5135	−0.0615	−0.3416	0.065*	
H16	0.6296	0.0073	−0.3332	0.065*	
C18	0.5219 (3)	0.26959 (15)	0.0045 (3)	0.0323 (5)	0.77 (1)
H17	0.5606	0.3273	0.0118	0.039*	0.77 (1)
H18	0.4387	0.2669	−0.0784	0.039*	0.77 (1)
C19	0.4804 (4)	0.25264 (16)	0.1230 (3)	0.0594 (12)	0.77 (1)
H19	0.4462	0.1946	0.1185	0.071*	0.77 (1)
H20	0.4069	0.2920	0.1222	0.071*	0.77 (1)
H21	0.5608	0.2602	0.2058	0.071*	0.77 (1)
C18B	0.5542 (8)	0.2758 (5)	0.0648 (8)	0.0266 (14)	0.23 (1)
H18B	0.6088	0.2791	0.1621	0.032*	0.23 (1)
H17B	0.5549	0.3323	0.0256	0.032*	0.23 (1)
C19B	0.4075 (8)	0.2516 (5)	0.0447 (12)	0.051 (2)	0.23 (1)
H20B	0.3518	0.2518	−0.0513	0.062*	0.23 (1)
H21B	0.3689	0.2922	0.0910	0.062*	0.23 (1)
H19B	0.4062	0.1950	0.0812	0.062*	0.23 (1)
Cl1	0.81084 (3)	0.607708 (19)	−0.25218 (3)	0.03382 (12)	
O1	0.86688 (12)	0.31612 (6)	0.21685 (9)	0.0368 (2)	

### Atomic displacement parameters (Å^2^)

**Table:**

	*U*^11^	*U*^22^	*U*^33^	*U*^12^	*U*^13^	*U*^23^
C1	0.0290 (6)	0.0219 (5)	0.0220 (5)	−0.0018 (4)	0.0109 (5)	0.0022 (4)
C2	0.0254 (6)	0.0248 (5)	0.0172 (5)	−0.0015 (4)	0.0077 (4)	0.0017 (4)
C3	0.0321 (6)	0.0241 (5)	0.0211 (6)	−0.0001 (5)	0.0115 (5)	0.0006 (5)
C4	0.0350 (7)	0.0250 (6)	0.0222 (6)	−0.0062 (5)	0.0068 (5)	0.0025 (5)
C5	0.0245 (6)	0.0298 (6)	0.0406 (7)	−0.0038 (5)	0.0078 (6)	0.0082 (6)
C6	0.0290 (7)	0.0241 (6)	0.0435 (7)	0.0019 (5)	0.0163 (6)	0.0078 (5)
C7	0.0313 (6)	0.0241 (6)	0.0249 (6)	−0.0012 (4)	0.0149 (5)	−0.0013 (5)
C8	0.0228 (6)	0.0220 (5)	0.0232 (6)	−0.0011 (4)	0.0097 (5)	−0.0010 (4)
C9	0.0274 (6)	0.0227 (5)	0.0227 (6)	−0.0024 (4)	0.0092 (5)	−0.0032 (5)
C10	0.0262 (6)	0.0284 (6)	0.0215 (6)	−0.0013 (5)	0.0080 (5)	0.0000 (5)
C11	0.0199 (5)	0.0241 (5)	0.0281 (6)	0.0000 (4)	0.0067 (5)	0.0050 (5)
C12	0.0231 (6)	0.0214 (5)	0.0304 (6)	−0.0009 (4)	0.0079 (5)	−0.0032 (5)
C13	0.0242 (6)	0.0254 (6)	0.0236 (6)	−0.0012 (4)	0.0091 (5)	−0.0034 (5)
C14	0.0251 (6)	0.0299 (6)	0.0282 (6)	−0.0014 (5)	0.0097 (5)	0.0004 (5)
C15	0.0306 (7)	0.0426 (8)	0.0326 (7)	0.0021 (6)	0.0027 (6)	0.0042 (6)
C16	0.0452 (9)	0.0302 (7)	0.0377 (8)	−0.0135 (6)	0.0121 (7)	−0.0051 (6)
C17	0.0397 (8)	0.0535 (9)	0.0380 (8)	−0.0185 (7)	0.0164 (7)	−0.0165 (7)
C18	0.0272 (12)	0.0278 (9)	0.0408 (13)	0.0017 (8)	0.0109 (11)	0.0014 (11)
C19	0.086 (3)	0.0415 (14)	0.074 (2)	0.0227 (14)	0.057 (2)	0.0097 (13)
C18B	0.024 (4)	0.024 (3)	0.029 (3)	0.003 (2)	0.005 (3)	0.000 (3)
C19B	0.019 (3)	0.041 (4)	0.093 (7)	−0.010 (3)	0.020 (4)	−0.032 (4)
Cl1	0.03264 (19)	0.02795 (18)	0.03544 (19)	−0.00214 (11)	0.00560 (14)	0.01037 (12)
O1	0.0595 (7)	0.0309 (5)	0.0235 (5)	−0.0063 (4)	0.0192 (5)	−0.0014 (4)

### Geometric parameters (Å, °)

**Table:**

C1—C2	1.3988 (16)	C13—H6	0.968 (15)
C1—C6	1.4012 (18)	C14—C15	1.5292 (19)
C1—C7	1.5067 (16)	C14—H7	0.986 (17)
C2—C3	1.3956 (16)	C14—H8	1.007 (16)
C2—C14	1.5087 (17)	C15—H9	1.010 (19)
C3—C4	1.3849 (18)	C15—H10	0.976 (17)
C3—H1	0.969 (15)	C15—H11	0.975 (19)
C4—C5	1.3854 (19)	C16—C17	1.523 (2)
C4—C16	1.5115 (17)	C16—H12	0.957 (18)
C5—C6	1.3891 (19)	C16—H13	0.977 (19)
C5—H2	0.950 (19)	C17—H14	0.9800
C6—C18	1.503 (3)	C17—H15	0.9800
C6—C18B	1.649 (8)	C17—H16	0.9800
C7—O1	1.2181 (15)	C18—C19	1.500 (3)
C7—C8	1.4912 (16)	C18—H17	0.9900
C8—C9	1.3962 (16)	C18—H18	0.9900
C8—C13	1.3977 (16)	C19—H19	0.9800
C9—C10	1.3872 (17)	C19—H20	0.9800
C9—H3	0.919 (15)	C19—H21	0.9800
C10—C11	1.3862 (17)	C18B—C19B	1.502 (10)
C10—H4	0.946 (16)	C18B—H18B	0.9900
C11—C12	1.3891 (18)	C18B—H17B	0.9900
C11—Cl1	1.7387 (12)	C19B—H20B	0.9800
C12—C13	1.3807 (17)	C19B—H21B	0.9800
C12—H5	0.965 (16)	C19B—H19B	0.9800
			
C2—C1—C6	121.05 (11)	C2—C14—H7	109.7 (9)
C2—C1—C7	119.93 (11)	C15—C14—H7	108.7 (9)
C6—C1—C7	119.02 (11)	C2—C14—H8	107.9 (9)
C3—C2—C1	118.34 (11)	C15—C14—H8	110.9 (9)
C3—C2—C14	120.30 (11)	H7—C14—H8	107.2 (13)
C1—C2—C14	121.34 (11)	C14—C15—H9	111.0 (11)
C4—C3—C2	121.66 (11)	C14—C15—H10	110.6 (11)
C4—C3—H1	120.0 (9)	H9—C15—H10	107.7 (14)
C2—C3—H1	118.3 (9)	C14—C15—H11	112.2 (11)
C3—C4—C5	118.71 (11)	H9—C15—H11	108.4 (15)
C3—C4—C16	121.17 (13)	H10—C15—H11	106.8 (15)
C5—C4—C16	120.08 (12)	C4—C16—C17	112.27 (12)
C4—C5—C6	121.85 (12)	C4—C16—H12	109.7 (10)
C4—C5—H2	119.7 (10)	C17—C16—H12	109.1 (11)
C6—C5—H2	118.4 (10)	C4—C16—H13	108.9 (11)
C5—C6—C1	118.38 (12)	C17—C16—H13	110.6 (11)
C5—C6—C18	117.26 (14)	H12—C16—H13	106.1 (15)
C1—C6—C18	124.22 (14)	C16—C17—H14	109.5
C5—C6—C18B	129.1 (3)	C16—C17—H15	109.5
C1—C6—C18B	110.4 (3)	H14—C17—H15	109.5
O1—C7—C8	121.03 (11)	C16—C17—H16	109.5
O1—C7—C1	120.50 (11)	H14—C17—H16	109.5
C8—C7—C1	118.45 (10)	H15—C17—H16	109.5
C9—C8—C13	119.41 (11)	C19—C18—C6	112.02 (19)
C9—C8—C7	121.38 (10)	C19—C18—H17	109.2
C13—C8—C7	119.21 (10)	C6—C18—H17	109.2
C10—C9—C8	120.42 (11)	C19—C18—H18	109.2
C10—C9—H3	118.4 (9)	C6—C18—H18	109.2
C8—C9—H3	121.2 (9)	H17—C18—H18	107.9
C11—C10—C9	118.79 (11)	C19B—C18B—C6	111.8 (5)
C11—C10—H4	120.8 (9)	C19B—C18B—H18B	109.3
C9—C10—H4	120.4 (9)	C6—C18B—H18B	109.3
C10—C11—C12	121.96 (11)	C19B—C18B—H17B	109.3
C10—C11—Cl1	119.08 (9)	C6—C18B—H17B	109.3
C12—C11—Cl1	118.94 (9)	H18B—C18B—H17B	107.9
C13—C12—C11	118.65 (11)	C18B—C19B—H20B	109.5
C13—C12—H5	121.5 (9)	C18B—C19B—H21B	109.5
C11—C12—H5	119.9 (9)	H20B—C19B—H21B	109.5
C12—C13—C8	120.76 (11)	C18B—C19B—H19B	109.5
C12—C13—H6	121.6 (8)	H20B—C19B—H19B	109.5
C8—C13—H6	117.7 (8)	H21B—C19B—H19B	109.5
C2—C14—C15	112.32 (11)		
			
C6—C1—C2—C3	−0.82 (17)	C1—C7—C8—C9	−13.65 (17)
C7—C1—C2—C3	178.67 (10)	O1—C7—C8—C13	−12.02 (18)
C6—C1—C2—C14	−179.45 (11)	C1—C7—C8—C13	166.36 (11)
C7—C1—C2—C14	0.04 (16)	C13—C8—C9—C10	−0.16 (18)
C1—C2—C3—C4	0.12 (17)	C7—C8—C9—C10	179.84 (11)
C14—C2—C3—C4	178.77 (11)	C8—C9—C10—C11	−0.69 (18)
C2—C3—C4—C5	0.22 (18)	C9—C10—C11—C12	0.58 (18)
C2—C3—C4—C16	177.85 (11)	C9—C10—C11—Cl1	179.47 (9)
C3—C4—C5—C6	0.13 (19)	C10—C11—C12—C13	0.41 (18)
C16—C4—C5—C6	−177.53 (13)	Cl1—C11—C12—C13	−178.49 (9)
C4—C5—C6—C1	−0.8 (2)	C11—C12—C13—C8	−1.29 (18)
C4—C5—C6—C18	175.11 (16)	C9—C8—C13—C12	1.18 (18)
C4—C5—C6—C18B	−162.6 (4)	C7—C8—C13—C12	−178.83 (11)
C2—C1—C6—C5	1.15 (19)	C3—C2—C14—C15	−91.80 (14)
C7—C1—C6—C5	−178.34 (11)	C1—C2—C14—C15	86.80 (14)
C2—C1—C6—C18	−174.45 (16)	C3—C4—C16—C17	−92.27 (16)
C7—C1—C6—C18	6.1 (2)	C5—C4—C16—C17	85.33 (16)
C2—C1—C6—C18B	166.2 (3)	C5—C6—C18—C19	80.7 (3)
C7—C1—C6—C18B	−13.3 (3)	C1—C6—C18—C19	−103.7 (2)
C2—C1—C7—O1	−88.68 (15)	C18B—C6—C18—C19	−47.5 (8)
C6—C1—C7—O1	90.82 (16)	C5—C6—C18B—C19B	−9.0 (8)
C2—C1—C7—C8	92.93 (14)	C1—C6—C18B—C19B	−172.0 (6)
C6—C1—C7—C8	−87.57 (14)	C18—C6—C18B—C19B	55.1 (9)
O1—C7—C8—C9	167.97 (12)		
